# The Dfam database of repetitive DNA families

**DOI:** 10.1093/nar/gkv1272

**Published:** 2015-11-26

**Authors:** Robert Hubley, Robert D. Finn, Jody Clements, Sean R. Eddy, Thomas A. Jones, Weidong Bao, Arian F.A. Smit, Travis J. Wheeler

**Affiliations:** 1 Institute for Systems Biology, Seattle, WA 98109, USA; 2 European Molecular Biology Laboratory, European Bioinformatics Institute (EMBL-EBI), Wellcome Trust Genome Campus, Hinxton, Cambridge, CB10 1RQ, UK; 3 HHMI Janelia Research Campus, Ashburn, VA 20147, USA; 4 Howard Hughes Medical Institute, Harvard University, Cambridge, MA 02138, USA; 5 Genetic Information Research Institute, Los Altos, CA 94022, USA; 6 University of Montana, Missoula, MT 59812, USA

## Abstract

Repetitive DNA, especially that due to transposable elements (TEs), makes up a large fraction of many genomes. Dfam is an open access database of families of repetitive DNA elements, in which each family is represented by a multiple sequence alignment and a profile hidden Markov model (HMM). The initial release of Dfam, featured in the 2013 NAR Database Issue, contained 1143 families of repetitive elements found in humans, and was used to produce more than 100 Mb of additional annotation of TE-derived regions in the human genome, with improved speed. Here, we describe recent advances, most notably expansion to 4150 total families including a comprehensive set of known repeat families from four new organisms (mouse, zebrafish, fly and nematode). We describe improvements to coverage, and to our methods for identifying and reducing false annotation. We also describe updates to the website interface. The Dfam website has moved to http://dfam.org. Seed alignments, profile HMMs, hit lists and other underlying data are available for download.

## INTRODUCTION

Annotation of the repetitive content of a genome depends on the initial discovery of repeat families present in that genome (so called *de novo* identification, e.g. (1–3)), followed by homology-based annotation (4), in which tools are used to seek all recognizable members of those families. The purpose of Dfam is to improve this homology-based annotation step, using profile hidden Markov models (profile HMMs) to improve detection of remote homologs of known families. Increased sensitivity is vital, as copies of older transposable elements (TEs) can be exceptionally difficult to detect because of accumulated mutations.

For each TE family, Dfam contains a multiple sequence alignment and a profile HMM constructed from that alignment. Profile HMMs (5,6) are probabilistic models that capture position-specific conservation information (7–10). The profile HMM search tool *nhmmer* (11) has been incorporated as a search engine for RepeatMasker (http://repeatmasker.org), so that the Dfam profile library can be used by RepeatMasker to increase the amount of genomic sequence that can be identified as derived from TE activity. The entries in Dfam are designed to be a drop-in replacement for the Repbase-derived library of consensus sequences (12) used by RepeatMasker for repeat detection. In this release, we see an increase in repeat annotation coverage, relative to annotation using RepeatMasker with the Repbase-derived library and cross_match search tool (http://www.phrap.org/phredphrapconsed.html). The increase in the fraction of the genome thus annotated is +5.1% for human, 5.5% for mice, 4.4% for zebrafish, 0.7% for flies and 6.5% for nematodes. In expanding Dfam to include families from multiple genomes, we selected the four species named above because of their status as model organisms covering a broad range of the animal kingdom. Over time, the Dfam library will continue to grow to include repeat families for other organisms. This will be done via a combination of building upon the mature collection of TE families in Repbase, and providing curation tools to facilitate the acquisition of new entries from the wider scientific community.

## NEW GENOMES

Dfam 2.0 contains all repeat families represented in Repbase (20.07) and RepeatMasker (20150807) databases for 5 representative species: *H. sapiens* [hg38], *M. musculus* [mm10], *D. rerio* [danRer10], *D. melanogaster* [dm6] and *C. elegans* [ce10]. Each family is built around a multiple alignment of representative sequences. Each of these *seed alignments* is based on the Repbase-derived RepeatMasker library of consensus sequences, leveraging the substantial effort that has gone into defining consensus sequences for the numerous repeat families. Each family in Dfam is assigned to one or more clades in the NCBI taxonomy (13). This clade contains all the descendants of the organism in which the element was active, or which obtained the repeat through horizontal transfer. Thus some recently derived TEs are associated with a single species, while others may be associated with a broader group. For example MIR (DF0000001), the ‘Mammalian-wide Interspersed Repeat’, is assigned the taxon Mammalia, and is found in both humans and mice.

Dfam families include retrotransposons, DNA transposons, interspersed repeats of unknown origin, and a number of non-TE entries used to annotate satellites or to avoid annotating noncoding RNA genes as TEs. The distribution of these constituent family types is given in Table 1.

**Table 1.** Composition of Dfam. In addition to the repeat families represented here, Dfam contains 76 non-coding RNA families and 92 satellite families

	Retrotransposons	DNA transposons	Unknown origin
Human only	428	46	1
Mouse only	544	9	8
All mammals	388	277	62
Zebrafish	1074	766	13
Fly	165	27	10
Nematode	57	98	8

## SEED ALIGNMENT CONSTRUCTION

Our techniques for constructing seed alignments for each family have advanced since the initial Dfam release. Each seed alignment consists of up to 2000 sequences belonging to that family. Family membership and sequence boundaries are determined by RepeatMasker with its most sensitive settings, using the consensus sequence of the family (from Repbase), and cross_match. If more than 2000 instances are available, instances in the most divergent quartile are removed and 2000 from the remaining set are chosen randomly. Alignments covering over 75% of the consensus length are used before shorter fragments are considered. If regions with low coverage remain, 10x coverage at each position is achieved (if possible) by adding instances from another source organism (e.g. alligator, platypus). For each sequence, the alignment against the consensus is provided by RepeatMasker; these sequences are joined into a multiple sequence alignment based on their alignments to the shared consensus.

For Dfam2, construction of seed alignments was done in this way to make maximal use of existing high-quality curated families. The RepeatMasker alignments have the following advantages: (i) There are very few false positives or false extensions into unrelated DNA. (ii) RepeatMasker excises simple repeat expansions and insertions of younger TEs, so that more and longer uninterrupted instances of underlying TEs can be recognized and included in the seed. (iii) RepeatMasker uses directional alignment parameters (gap penalties and log-odds substitution matrices) accurately reflecting isochore-specific neutral decay patterns from an original sequence (the consensus) to the current state of copies. (iv) A single genomic sequence may be matched by two or more family searches, because many TEs are related to each other. We call these redundant hits. By letting RepeatMasker pick the best from among those redundant hits, we largely avoid assigning such a sequence to the wrong seed alignment. However, Dfam seed alignments are not required to be derived from RepeatMasker annotation. In the future, some seed alignments will be constructed directly during the family curation process, rather than depending on a consensus sequence and RepeatMasker run.

Even when using RepeatMasker alignments based on curated consensus sequences, multiple issues can lead to suboptimal seed alignments, especially for lower copy elements. In these cases, some intervention is required. (i) Copies amplified through tandem or segmental duplications long after the activity of the transposable elements can skew the profile of a family, particularly if few copies are available overall. To reduce this problem, in any case where there are instances that have >90% similarity to each other over 250 bp of flanking DNA on either side, we select only one representative copy. (ii) The repeat databases, even for human, are far from complete and a genome harbors many copies of unrepresented elements that may be (partially) matched by those in the database in the RepeatMasker analysis. Inclusion of these matches dilutes the signal for the intended element. The fact that these cross-matches tend to show higher divergence from the consensus motivates the previously mentioned exclusion of the most divergent sequences from the seed. (iii) Another problem can arise when a low copy number element contains similarity to a high copy element, e.g. a complex repeat like SVA including an Alu. Even if RepeatMasker mistakenly annotates only a very small fraction of the high copy element as a fragment of the low copy element, this small fraction may overwhelm the count of true instances in that region of the seed alignment. When this seed is used to search the genome, the signal induced by these mistakenly included sequences may exacerbate the problem of wrongly annotating members of the high copy family. Since these matches are only to a part of the consensus or model, sequences covering only those regions where the consensus matches a more common repeat are excluded from the seed alignment. This last method has not yet been automated, and has been applied to a few families by manually removing incorrectly included sequences from the seed.

In Dfam 1.0, all sequences in the seed alignment were required to come from the human genome. A major improvement in the quality of the HMMs in Dfam 2.0 derives from the ability to build seed alignments not only from instances in the target genome but from any other genome containing copies of the same TE. For example, copies of an element active before the eutherian radiation are present in a reconstructed eutherian ancestral genome (14,15), in which far fewer substitutions have accumulated. Likewise, the highly diverged copies in mouse of TEs that were active before the rodent-primate split, are present relatively intact in the human genome, as the neutral decay rate in primates has been much lower than in rodents. Models for such old repeats constructed from human alignments performed better in mouse, both with respect to sensitivity and selectivity, than models constructed from mouse-only copies (data not shown). In the current release, for models with incomplete seed coverage, we chose to use related but slower evolving species to generate the seed. As an example, alligator instances were used to supplement several amniote-wide repeats. For the zebrafish, fruit fly and nematode, all models were built from native sequences, as no genomes of slower evolving close relatives or reconstructed ancestors exist as yet.

## GENOME ANNOTATION

When annotating a genome with Dfam, two important issues should be considered: (i) Redundant hits arise when more than one family matches a single genomic sequence; a tool must compare such redundant hits, and assign the ‘best” TE classification to each region. (ii) Young TEs often insert in the middle of an already-present TE. We should account for these, for example by identifying and extracting the younger inserts, then searching remaining sequence for older TEs. We recommend using RepeatMasker for annotation, as it has incorporated Dfam and *nhmmer*, while also handling these concerns. For the annotation of mammalian genomes with Dfam models, RepeatMasker first identifies and clips out near-perfect simple tandem repeats, using TRF (16), then follows a multi-stage process designed to ensure accurate annotation of possibly-nested repeats. For non-mammals, the TRF step is followed by only a single excision and masking pass of all repeats.

In all cases, Dfam models are searched against the target genome using model-specific score thresholds described later. The format of RepeatMasker's Dfam-based output is nearly identical to the traditional cross_match-based output, with cross_match type alignments of copies to consensus sequences extracted from the HMMs. As a matter of convenience, we also provide a simplistic script, called dfamscan.pl, to address redundant hits.

## SENSITIVITY AND FALSE ANNOTATION; BENCHMARKS AND IMPROVEMENTS

Our analyses with the initial release of the database (17) found increased coverage by profile HMMs relative to their consensus counterparts, while simultaneously maintaining a low false discovery rate. For this release we have further developed methods for benchmarking the specificity and sensitivity of the models. To assess specificity, we developed two benchmarks, one designed to identify the rate of false positive hits, and the other designed to identify cases of overextension. In overextension, a hit correctly matches a truncated true instance but then extends beyond the bounds of that instance into flanking non-homologous sequence (18). We define coverage to be the number of nucleotides in real genomic sequence that are annotated by the search method. Assuming the benchmarks correctly suggest the rate of false coverage, sensitivity is the genomic coverage minus false coverage. Using these new benchmarks we were able to identify areas for improvement in the model building processes. Here we describe our new benchmarks, approaches we have used to reduce false annotation, and the impact on annotation.

### New benchmark for false positives

We use a synthetic benchmark dataset to estimate false positive hit rates and to establish family-specific score thresholds, which indicate the level of similarity required to be considered safe to annotate. Until Dfam 1.3, we used reversed, non-complemented sequences as our false positive benchmark, as this appeared to be the most challenging (i.e. produced the most false positives) of the method we tested with TE identification algorithms. Starting with Dfam 1.4 we switched to a new benchmark, using simulated sequences that display complexity comparable to that seen in real genomic sequence. These sequences are simulated using GARLIC (19), which uses a Markov model that transitions between six GC content bins, basing emission probability at each position on the most recently-emitted three letters (a fourth-order Markov model). After constructing such sequences, GARLIC inserts synthetically diverged instances of simple repeats based on the observed frequency of such repeats in real genomic GC bins. Sequences produced by GARLIC more accurately match the distributions of k-mers found in real genomic sequence, and are a more stringent benchmark (produce more false hits) than other methods tested, including reversed genomic sequence.

As in previous Dfam releases, the false positive benchmark is used to establish score thresholds for each model. The ‘gathering’ (GA) threshold is to be applied when the family is known to exist in the annotated organism, and ensures high sensitivity with a low frequency of false positives among annotated sequences. For example, a family profile may have a mouse-specific GA threshold, which should be used in annotating members of that family in the mouse genome. The ‘trusted cut-off’ (TC) threshold is more stringent, and is intended for use when annotating other organisms. When searching Dfam models with *nhmmer*, the GA threshold is accessed using the flag ‘- -cut_ga’, and the TC threshold is accessed using ‘- -cut_tc’.

For each family, thresholds were established for each Dfam organism known to contain instances of that family. All models were searched against that organism's genomic sequence, and also against a simulated GARLIC genome of the same size. All new models were searched with an *E*-value cut-off of 100. The GA threshold was chosen to ensure an empirical false discovery rate of ≤0.2% and maximum *E*-value of ≤100. The GARLIC hit count is assumed to represent the number of false hits on genomic sequence, and false discovery rate (FDR) is the percent of all genomic hits that are false hits; see (17). When there are <50 000 true hits in the family, FDR = 0.2% dominates; for very high count families, the *E*-value threshold will limit accepted false annotation. The TC threshold is at least as high as necessary to reach an E-value of 0.0001 for that model, and is adjusted upwards so that it is always higher than any false hit on the GARLIC sequence (i.e. an empirical FDR of ∼0).

### Overextension

We developed a related benchmark to assess overextension behavior. Our benchmark uses GARLIC to place truncated and mutated instances of known TEs into simulated background. We expect matches to these planted instances, and any expansion of alignments into the flanking simulated sequence can be identified as overextension. This benchmark highlighted the fact that false extensions were a greater concern than we previously reported.

Many repeat families demonstrate non-random patterns of association with particular composition landscapes (isochores). For example, L1s are usually located within AT-rich regions (20,21). If a true L1 fragment is found within an AT-rich region of the profile, and the flanking unaligned portion of the query is also AT-rich, a sequence alignment method may be lured into extending into that non-homologous flanking sequence, not because of homology, but because of composition. In (17), we assessed overextension by interleaving true repeats with reversed genomic sequence, without regard for the flanking composition. This led to an underestimate of the overextension problem. GARLIC inserts repeat copies preferentially into regions of GC content similar to those in which they most often occur, and it is this pattern that seems to most strongly induce overextension in *nhmmer*. Similar indications of overextension (not shown) were seen in a benchmark with design much like that in (17), but where repeat copies were placed in reversed sequence in precisely the same position in which they occurred in unreversed sequence (i.e. the surrounding sequence was now a false positive, but the bounding GC content was precisely the same as it was in unreversed sequence).

### Reducing overextension by increasing average relative entropy

**Figure 1.**
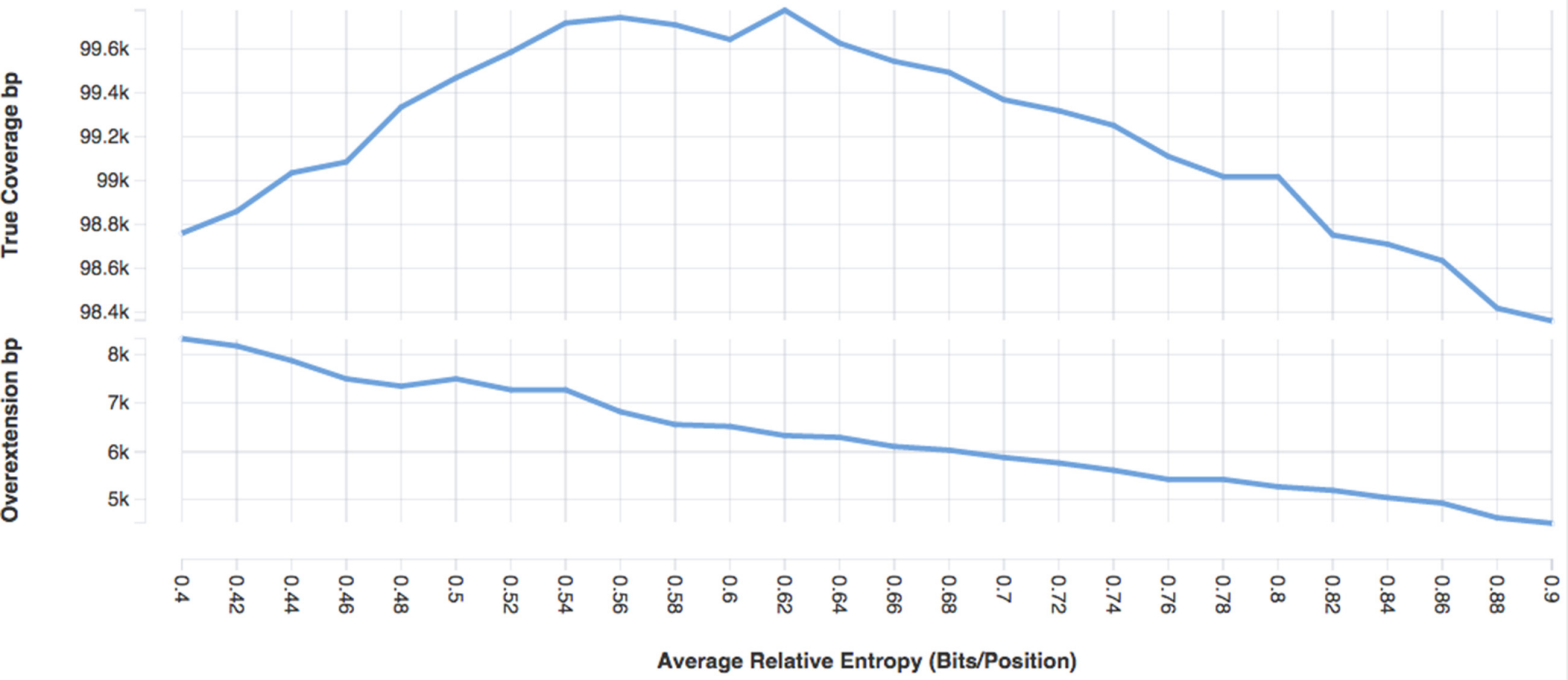
Influence of average relative entropy on annotation for one family. This plot shows the impact of target average relative entropy values of the Charlie15a (DF0000089) model on both annotation coverage (true positives) and overextension. Using the Charlie15a seed, profile HMMs were built with HMMER's *hmmbuild* tool, with varying target average relative entropy values ranging from 0.4 to 0.9 bits per position, using the - -ere flag. The largest of these values represents the average relative entropy of the model when no sequence downweighting (entropy weighting) is performed. Coverage was assessed by searching each entropy-weighted profile HMM against the human genome. Overextension was assessed by searching each profile against a simulated genome containing fragments of true Charlie15a elements planted into realistic simulated genomic sequence built using GARLIC.

In the context of sequence alignment, relative entropy (22,23) amounts to the expected score of an aligned character. Lower relative entropy corresponds to increased divergence among sequences. For single-sequence comparison methods like cross_match and blast (8), a single scoring matrix is applied across the entire alignment; expected scores do not vary from position to position. Low relative entropy substitution matrices have been shown to permit high levels of overextension (24,25), and low relative entropy has a similar impact in the context of profile hidden Markov model alignment (Rivas & Eddy, submitted). Previously, *nhmmer* aimed to construct profile HMMs with a target average relative entropy of 0.45 bits per position; raising this default to 0.62 bits/position did not greatly detract from hit sensitivity, but did reduce levels of overextension. An example of the impact of target relative entropy on the sensitivity and overextension of one repeat family is given in Figure 1. The impact of relative entropy on overall human coverage is shown in Table 2.

**Table 2.** Influences of average relative entropy on annotation for all human families

Average relative entropy	Overextension change (bp)	True positive change (bp)
0.40	1 187 973	(472 522)
0.42	686 251	(255 520)
0.44	242 979	(43 430)
0.46	(242 979)	43 430
0.48	(717 069)	119 880
0.50	(1 160 532)	200 545
0.52	(1 586 452)	104 369
0.54	(2 001 938)	(2416)
0.56	(2 440 201)	(121 426)
0.58	(2 812 553)	(287 361)
0.60	(3 236 722)	(586 724)
0.62	(3 624 644)	(873 070)
0.64	(3 976 080)	(1 264 674)
0.66	(4 326 797)	(1 629 825)
0.68	(4 670 024)	(2 011 169)
0.70	(4 974 778)	(2 462 143)

Using the GARLIC benchmark with inserted TE fragments, we tested a variety of target average relative entropy values, assessing the impact on coverage and overextension across all human models. Values in parentheses are negative, indicating a reduction in overextension or coverage from the previous default of 0.45 bits per position. We chose to update the default in HMMER to a higher value (0.62) to reduce overextension while only sacrificing a modest amount of true positive matches.

### Position specific entropy weighting to reduce overextension

In seed alignments, some columns are more conserved than others. More-conserved columns have higher relative entropy than less-conserved columns. Moreover, these alignments often show variability in coverage—some columns are represented by many sequences, while others are only represented by a few. This is particularly true in families where few full-length copies are known. When computing a profile HMM from a seed alignment, HMMER mixes observed counts with a prior distribution; more observed counts means less reliance on the prior, and (on average) greater relative entropy. Thus Dfam's profile HMMs demonstrate position-specific variability in relative entropy due to a combination of the number of observations in a column and the conservation within those observations.

By default, the average (per-position) relative entropy of a model, after mixing observed counts with the prior model, might be much higher than the target average relative entropy (0.62 bits per position). HMMER achieves the target value by down-weighting the number of observations, in a process called *entropy weighting* (9). This essentially increases the influence of the prior. The default in HMMER is to uniformly down-weight observations in all columns by a multiplicative factor, picking a factor that causes the target to be reached. We found that this can be problematic in the case of very fragmented Dfam seed alignments, in which there can be high variability in column coverage. For columns with relatively few observations, the uniform multiplier can lead to unreasonably small (adjusted) observations. This is common, for example, due to the pervasive 5’ truncation of LINE copies, where observed counts in one part of the seed can be more than an order of magnitude smaller than in another. Similar to observations of high overextension under low relative entropy scoring schemes, we found that Dfam overextension preferentially occurs in hits that end in these regions of low local relative entropy (data not shown).

**Figure 2.**
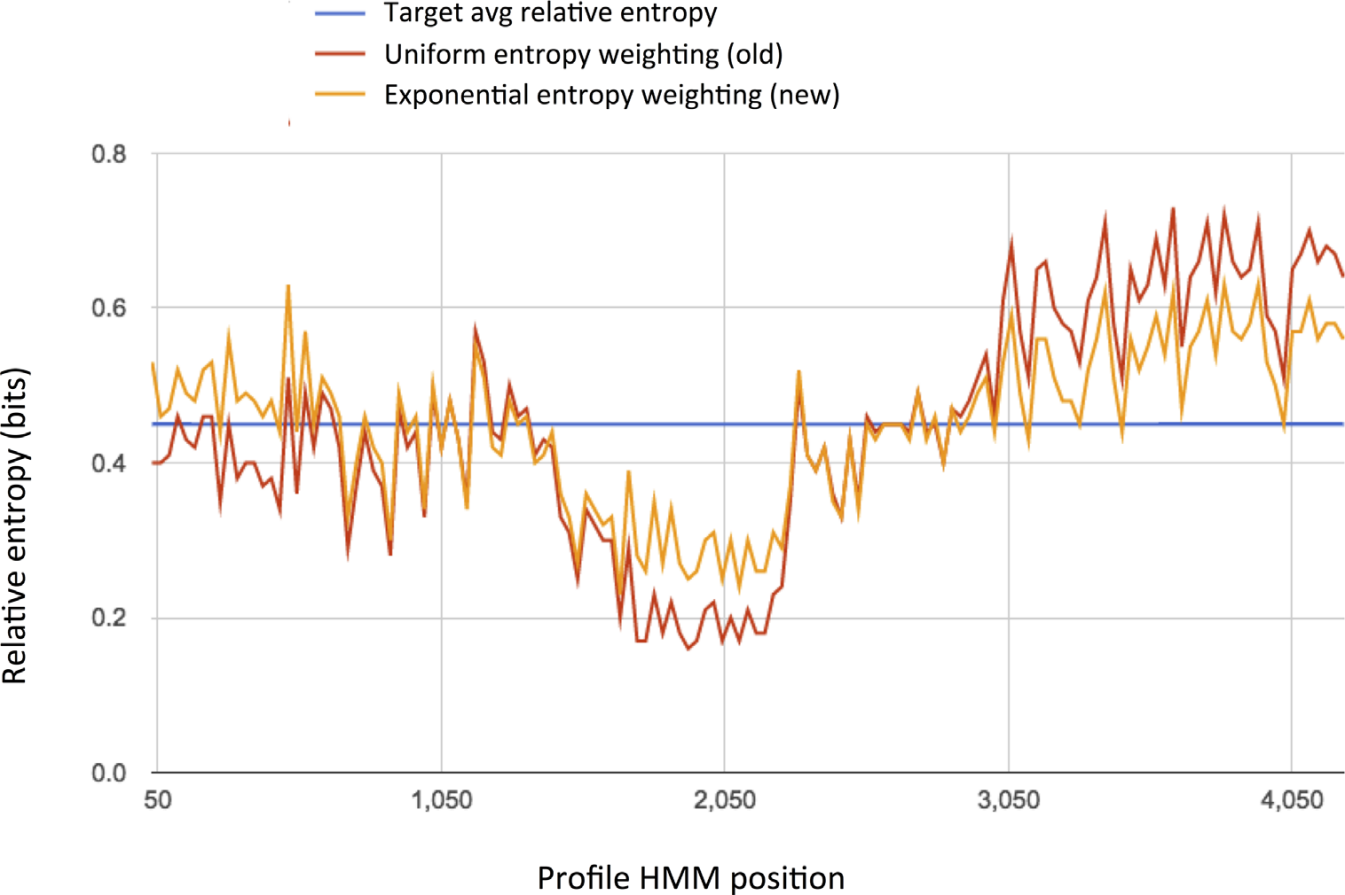
Impact of exponential entropy weighting on position-specific relative entropy. L1PREC2_5end (DF0000315) per-position relative entropy averaged over 30 bp windows with uniform and exponential entropy weighting functions. The region around position 1900 caused both false hits and overextension of true hits when using uniform entropy weighting; most of these were removed with the higher positional relative entropy generated using exponential entropy weighting.

Beginning with the Dfam 1.4 release, we devised a new scaling approach, which reduces the relative entropy of regions with higher coverage to a greater extent than those with lower coverage. Rather than finding a uniform multiplier, this method identifies an exponential scaling factor *s* that leads to the target relative entropy. Suppose a column has k observed letters; the scaled count will be *k^s^*. This factor 0 ≤ *s* ≤ 1 is applied to each column. Each scaled count will be between 1 and the true observed count *k*, and columns with low *k* are less dramatically down-weighted. This weighting variant is the new - -eentexp flag in *nhmmer*. See Figure 2 for an example of the impact of this approach on position-specific relative entropy. Employing the exponential weighting function on the Dfam seed alignments led to a decrease in overextension of hits for many models.

**Figure 3.**
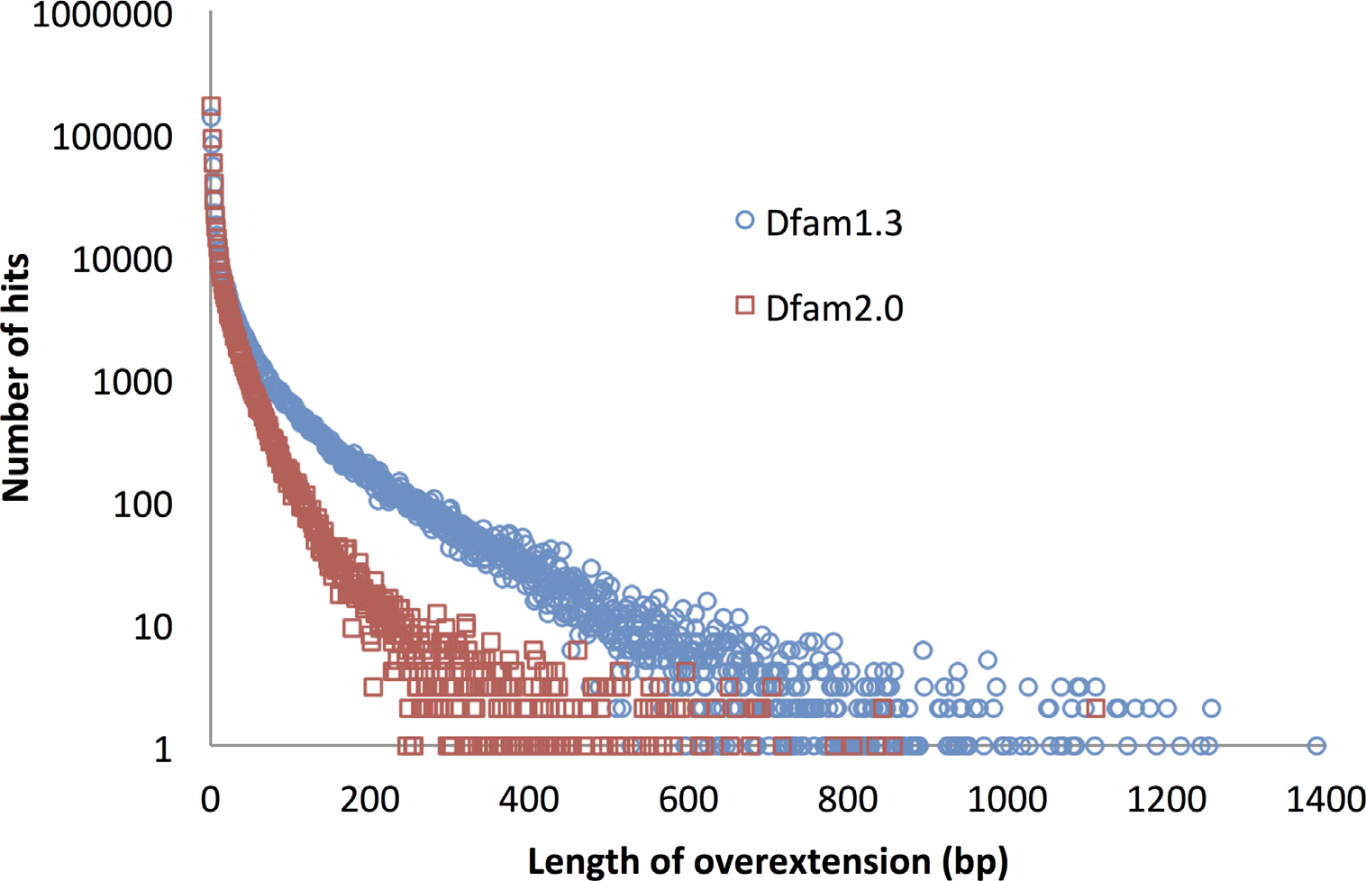
Distribution of overextension lengths. Profile HMMs for human Dfam families were searched against an overextension benchmark trained on human sequence data, built using GARLIC. For each hit above GA threshold, overextension was calculated. The plot shows, for each overextension length, the number of hits with that length. Application of our two changes (increased average relative entropy and exponential entropy weighting) clearly reduced the frequency of very long overextensions.

We evaluated the new Dfam release, based on these two changes in relative entropy calculation (target level, entropy weighting) using a GARLIC benchmark sequence and found the false discovery rate to be more than halved (Table 3). Even these rates are likely an overestimate of the true overextension FDR, since the benchmark contains fragmentary TE instances, while full length instances in real genomic sequence can not be overextended. Importantly, 75% of the improvement in overextension came from the elimination of long (>100 bp) overextensions (Figure 3).

**Table 3.** Improvements to false annotation

	FDR due to false hits	FDR due to overextension
*cross_match + consensus*	0.12%	1.09%
*nhmmer + Dfam 1.3*	0.63%	5.74%
*nhmmer + Dfam 2.0*	0.17%	2.34%

We used RepeatMasker to search the full set of human families against (i) the human genome (to count annotation coverage) and (ii) a GARLIC overextension benchmark based on simulated human genome sequence (to assess false coverage and overextension). This is a pessimistic estimate of the overextension FDR. RepeatMasker was tested with cross_match (v 1.080812) and the Repbase-derived RepeatMasker library (20.07, 20150807), and using nhmmer to search with both Dfam 1.3 profile models and Dfam 2.0 models.

### Overall annotation results

Dfam and *nhmmer* have been incorporated into RepeatMasker, and were used to annotate the five represented genomes: human [hg38], mouse [mm10], zebrafish [danRer10], fly [dm6], and nematode [ce10]. Validation using our benchmark indicates that false annotation can be kept low while retaining a high coverage. Specifically, Tables 4 and 5 demonstrate the gains in annotation coverage of Dfam relative to the prevalent method of annotating based on alignment to consensus sequences from the Repbase-derived RepeatMasker library.

**Table 4.** Increase in number of annotated interspersed repeats, using Dfam + nhmmer

		Interspersed repeats (count)	All repeats (count)	Increase (count)
Human	Consensus	3 743 222	4 532 893	
	Dfam	4 007 051	4 708 414	175 521
Mouse	Consensus	3 112 647	4 691 497	
	Dfam	3 640 848	5 152 510	461 013
Zebrafish	Consensus	2 757 001	3 577 995	
	Dfam	3 058 971	3 790 149	212 154
Fly	Consensus	22 819	119 232	
	Dfam	24 676	120 868	1636
Nematode	Consensus	46 566	80 064	
	Dfam	71 855	103 212	23 148

For each organism, RepeatMasker was run using (i) cross_match with consensus sequences from the Repbase-derived RepeatMasker library and (ii) nhmmer with Dfam2. Interspersed repeats are shown separately, while the all repeats count also includes locally repetitive satellites and short tandem repeats.

**Table 5.** Coverage gains for each organism, using Dfam + nhmmer

		Genome size (no Ns)	Interspersed repeats (bp)	All repeats (bp)	Increase (bp)	Increase (% of genome)
Human	Consensus	2 937 655 681	1 416 407 169	1 536 018 323		
	Dfam		1 570 963 110	1 686 550 773	150 532 450	5.1%
Mouse	Consensus	2 647 537 730	1 091 472 267	1 179 145 848		
	Dfam		1 234 765 488	1 324 034 627	144 888 779	5.5%
Zebrafish	Consensus	1 338 605 546	715 260 228	785 961 593		
	Dfam		781 171 892	844 630 681	58 669 088	4.4%
Fly	Consensus	137 077 099	20 456 863	25 281 658		
	Dfam		21 611 976	26 230 068	948 410	0.7%
Nematode	Consensus	100 286 070	10 245 891	12 532 750		
	Dfam		16 904 376	19 030 107	6 497 357	6.5%

RepeatMasker was used to search each organism as in Table 4. The genome size corresponds to assemblies hg38, mm10, danRer10, dm6 and ce10 respectively, in each case with _random, chrUn and _alt sequences removed and Ns ignored.

## NEW FEATURES ON THE WEBSITE

### Multiple species

**Figure 4.**
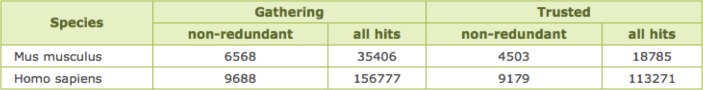
Hit statistics for MLT1A (DF0001126).

Changes to the Dfam website largely revolve around support for the presence of repeat families belonging to multiple species. The majority of the changes are on the back end of the website, involving speed and scalability. Here, we describe a few features that are visible on the website. On the Summary tab, the Hit Statistics section now provides observed hit counts for all appropriate species, as exemplified in Figure 4.

**Figure 5.**
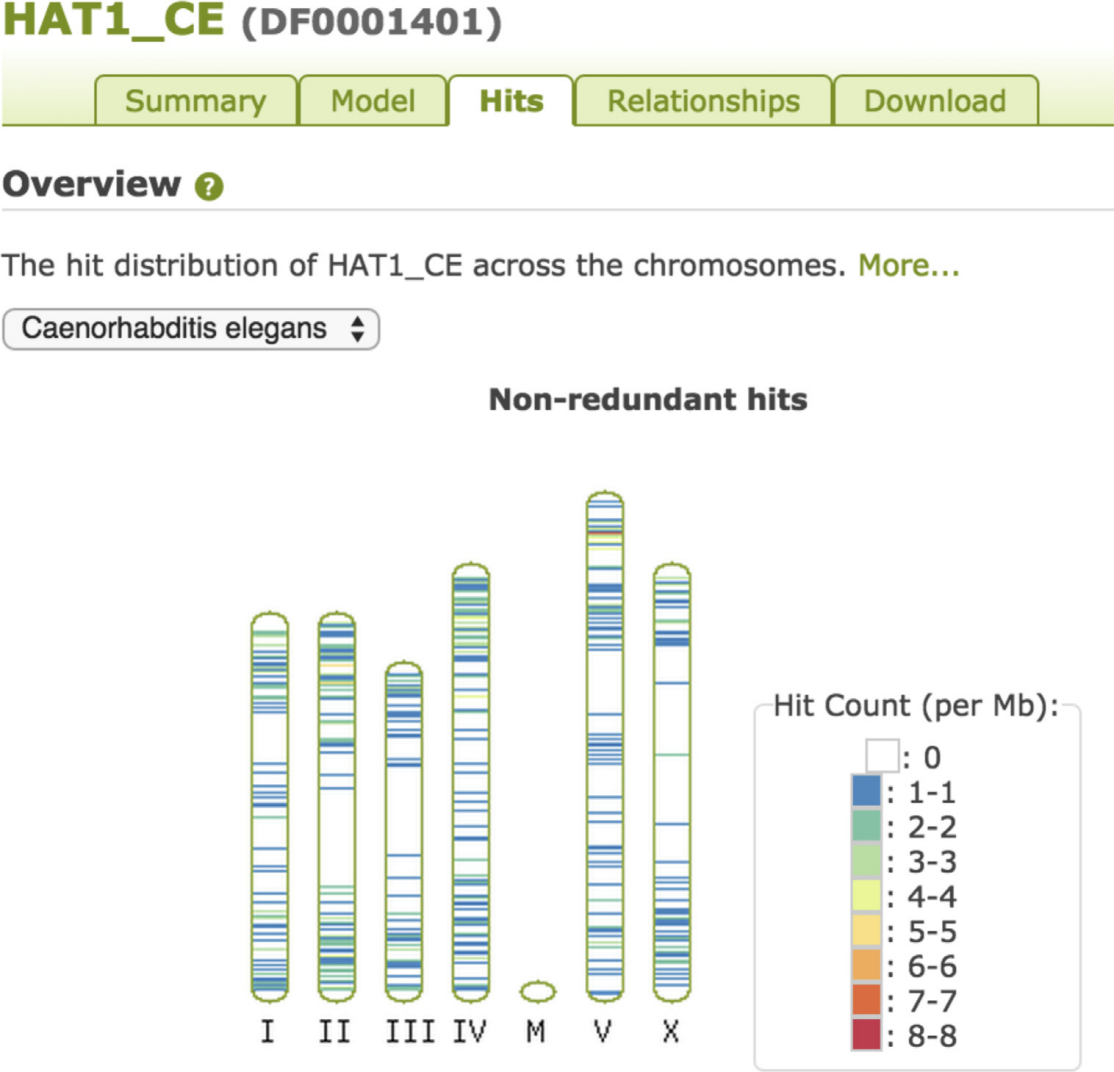
Hits displayed on karyotypes. This plot shows the distribution of HAT1_CE (DF0001401) elements across C. elegans chromosomes, demonstrating the well-known accumulation of some DNA transposons towards telomeres (26,27).

On the Model tab, the various coverage distribution plots are tied to the selection of an organism of interest, while general plots (such as those describing the seed alignment) are independent of this selection. Similarly, the Hits tab presents hits distributed across karyotype plots, depending on the selected organism, as seen in Figure 5. The Downloads tab also allows species-specific hit table downloads.

### New coverage plot

**Figure 6.**
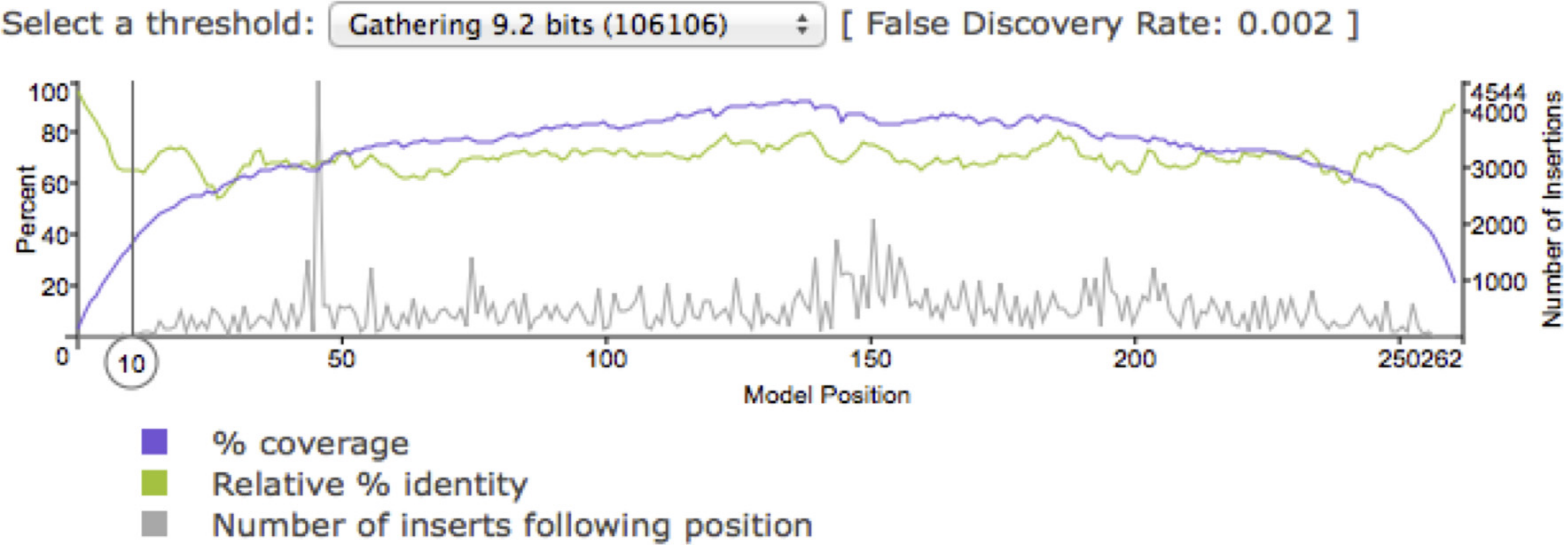
Coverage, Conservation, and Insert plot for MIR (DF0000001).

We have developed a new plot that compactly represents the distribution of hits along the family model according to a selectable score or E-value threshold. The plot also shows position-specific levels of conservation and insertion among those hits. It can highlight opportunities for improved curation or biologically interesting conservation patterns. For example, Figure 6 shows the plot for MIR, showing a steadily higher coverage in the center, which may be a consequence of an unresolved subfamily structure and/or of common exaptation of sequences in this ‘core domain’ (28–30).

## FUTURE CHALLENGES/DIRECTIONS

With this release, we have expanded the taxonomic coverage of Dfam, and expect that the increased annotation of the four new species will be a valuable addition. More importantly, we have begun to establish the framework for expansion to represent repetitive elements from across the tree of life. Over the coming years we will develop Dfam with two main approaches:

1. continue the protocol used here to build alignments and profile HMMs from the Repbase-derived RepeatMasker library, which contains consensus sequences for TEs from dozens of organisms;
2. build curation assistance tools to enable straightforward external contribution of families to the open-access Dfam database.

In order to support the species expansion in Dfam, we made significant changes to the database schema and middleware. Transposable elements can be tremendously prodigious, leaving millions of copies per element in a single genome; closely-related families result in redundant hit data. The tables storing the hits in Dfam1.0 contained over 110 million entries for the 1,143 families in human, and these numbers have grown to over 230 million entries for 4,150 total families in the current five organisms. In order to meet this scale, we refactored the schema to limit database tables to manageable scale, and optimized numerous data management scripts. Even so, expansion to repeat elements belonging to dozens or hundreds of organisms will overwhelm the current database format. We have begun development of more scalable options using a mix of relational and NoSQL database components. These will require further development, both in terms of technical architecture and overall framework for handling clade-specific repeats across the ever-growing collection of sequenced organisms.

Though changes to entropy weighting in *nhmmer* have substantially improved overextension behavior on our benchmarks, the problem is not solved. Additional methods are necessary to ensure that maximal sensitivity is retained, while further eradicating overextension. Another important source of false hits, discussed in the first Dfam paper, but still not resolved, is the handling of degenerate tandem repeats. Current methods involve masking both genomic sequence and family profile HMMs; new methods must be developed to directly model the existence of low complexity and tandemly repetitive sequence in genomic data.

## AVAILABILITY

The Dfam2 website site is available at http://dfam.org. Dfam data can be freely downloaded using the Download link at the top of every Dfam web page, either as flat files or in the form of MySQL table dumps. The Dfam database is supported by *nhmmer*, a part of HMMER3.1. A release snapshot of HMMER3.1, including the version of nhmmer used to produce the database and the results in this paper, is available via the same link. Data and scripts used to produce the various tables and figures can be downloaded at http://dfam.org/web_download/Publications/NAR2016/.
